# UAV-Based Safety-Oriented Object Detection for Live-Line Operations via Multi-Scale, Frequency-Aware, and State-Space Feature Enhancement

**DOI:** 10.3390/s26206598

**Published:** 2026-10-17

**Authors:** Pan Tang, Heyu Xiang, Fangqun Gao, Yuntao Wu, Yanduo Zhang, Tao Lu, Huabin Zhou, Md Zakaria Haque Sakib

**Affiliations:** 1 State Key Laboratory of Power Grid Environmental Protection, China Electric Power Research Institute, Wuhan 430074, China; tangpan178@gmail.com; 2 Hubei Key Laboratory of Intelligent Robot, School of Computer Science and Engineering, Wuhan Institute of Technology, Wuhan 430205, China; 22407010050@stu.wit.edu.cn (H.X.); zhangyanduo@hotmail.com (Y.Z.); lutxyl@gmail.com (T.L.); zhouhuabing@gmail.com (H.Z.); jakariashakib.bd@gmail.com (M.Z.H.S.); 3 Library, Wuhan Institute of Technology, Wuhan 430205, China

**Keywords:** UAV visual sensing, live-line operation, object detection, personal protective equipment, small-object detection, multi-scale feature fusion, frequency-aware representation, state-space model

## Abstract

Reliable visual sensing is important for UAV-assisted monitoring of live-line power operations, where workers, personal protective equipment (PPE), temporary insulation, and electrical structures appear under large-scale variation, occlusion, and background clutter. This study develops a safety-oriented detector that assigns different feature transformations to different stages of the detection hierarchy. Multi-Scale Selective Fusion (MSSF) combines 3-by-3, 5-by-5, and 7-by-7 receptive-field responses through channel-wise branch weighting. Frequency-Aware Adaptive Fusion (FreqAFU) refines the real and imaginary Fourier components and fuses the reconstructed response with the spatial feature through learned channel gates. BiNet and Mamba-based blocks provide bidirectional cross-scale interaction and state-space contextual modelling. The self-collected corpus contains 8124 annotated images and 118,119 object instances across eight categories, with 6499 training images and 1625 validation images separated at the acquisition-session level. On this fixed protocol, the complete YOLOv8n-based configuration improves mAP_50-95_ from 0.558 to 0.635 and mAP_50_ from 0.900 to 0.949. Ablations, repeated training, scale- and class-level analyses, runtime profiling, and VisDrone evaluation show that the benefit of individual components is configuration dependent rather than universally additive. The detector is positioned as an object-level perception layer; final safety-compliance reasoning remains outside its scope.

## 1. Introduction

Live-line operation is an important means of maintaining power distribution infrastructure without interrupting electricity supply. Because many tasks are performed at elevated positions and in close proximity to energized components, visual monitoring of worker protection and the surrounding operating environment is an important component of intelligent safety management. In typical high-altitude live-line operations, workers in bucket trucks wear insulating clothing, helmets, and insulating gloves, while temporary protective measures such as insulating covers and blankets are placed around potentially hazardous energized components. The surrounding pole and cross-arm structures provide additional spatial references for understanding the operating scene. Consequently, automated visual perception should not only identify the worker, but also jointly perceive the protective equipment, temporary insulation, and electrical structures that support subsequent safety-oriented analysis.

Unmanned aerial vehicles (UAVs) provide flexible, non-contact observation of live-line work sites and can capture elevated and oblique views that are difficult to obtain with fixed cameras. However, UAV imagery also introduces challenging visual conditions. A single image may contain a worker occupying only a small region, miniature protective equipment such as gloves and helmets, large elongated electrical structures, and temporary insulating materials with irregular shapes. Vegetation, construction equipment, shadows, and repeated textures further increase background interference. Changes in flight altitude and viewing angle produce large scale variations, while occlusion can hide part of a worker or protective equipment. These factors make conventional object detection methods less reliable for safety-critical visual targets.

The detection objective in live-line operation scenes also differs from conventional power-infrastructure inspection. The goal is not limited to identifying poles, conductors, or equipment defects. Instead, the detector should establish a structured representation of visible personnel, key personal protective equipment (PPE), temporary insulation measures, and electrical structural context. This object-level representation can subsequently support tasks such as PPE compliance analysis, insulation-state assessment, worker-to-structure spatial reasoning, and other rule-based or geometric safety evaluations. The detector is therefore regarded in this study as the perception layer of a broader safety-monitoring pipeline rather than as the final safety decision module.

Existing UAV detectors and remote-sensing detectors have made substantial progress in multi-scale feature fusion, attention-based refinement, and long-range context modeling [1,2,3,4,5,6]. Classical feature pyramids such as FPN, PANet, and EfficientDet established the importance of combining high-resolution localization cues with deeper semantic information [7,8,9]. Recent studies have further explored high-resolution representations, attention-assisted fusion, and context-guided small-object detection for remote-sensing imagery [10,11,12,13,14,15]. Nevertheless, three difficulties remain particularly relevant to live-line operation imagery. First, the receptive field required by a worker, a glove, and a pole may differ by a large margin, making fixed-scale aggregation suboptimal. Second, the boundaries of small PPE and insulating materials may be easily contaminated by high-frequency background textures, reducing localization quality. Third, the spatial relationships between a worker, protective objects, and electrical structures may extend across feature levels and spatially distant regions. These observations motivate a detector that explicitly combines scale adaptation, structural refinement, and contextual interaction.

Frequency-aware representations offer a complementary way to preserve structural information under cluttered backgrounds [16,17]. Long-range contextual modeling has traditionally relied on self-attention [18], while structured state-space models provide an alternative for efficient sequence interaction [19,20,21]. Recent power-oriented studies further show the practical value of UAV-based protection-object detection and safety-related scene analysis [22,23,24].

In this work, we develop a safety-oriented UAV object detection framework for live-line operations. The design is motivated by the observation that the dominant representation difficulties are distributed across the feature hierarchy rather than concentrated at a single stage. Small PPE can be weakened during downsampling, repeated background structures can interfere with local boundary responses, and the scene context relating personnel, protective objects, and electrical structures spans different feature levels and spatial regions. The proposed architecture therefore assigns different transformations to different stages instead of stacking all enhancement operations at one location.

Specifically, Multi-Scale Selective Fusion (MSSF) is introduced within feature extraction to preserve and adaptively combine responses from different receptive fields. Frequency-Aware Adaptive Fusion (FreqAFU) refines the real and imaginary Fourier components and regulates their contribution to the spatial representation through learned channel gates. BiNet establishes reciprocal top-down and bottom-up feature interaction, while Mamba-based state-space blocks are used on compact semantic feature maps to incorporate broader contextual information. The methodological contribution is thus defined by the task-oriented allocation and interaction of these operations within the detection hierarchy, rather than by claiming the underlying convolution, Fourier transform, bidirectional fusion, or state-space operator as a new primitive.

A dedicated UAV dataset was collected from live-line operation scenes. It contains 8124 annotated images and 118,119 object instances in eight semantically organized categories: person, insulated cover, insulated blanket, insulated clothing, insulated gloves, helmet, telegraph pole, and cross arm. These classes are selected according to their roles in safety-oriented perception. The person class denotes visible personnel who can serve as candidate worker instances for downstream association; helmet, insulated clothing, and insulated gloves represent key PPE; insulated cover and insulated blanket represent temporary insulation measures; and telegraph pole and cross arm provide electrical structural context for downstream spatial reasoning. The dataset therefore targets a specific safety-perception problem rather than attempting to reproduce the broad label space of generic UAV benchmarks.

The main contributions are summarized as follows:

- A task-oriented feature-enhancement architecture is developed for UAV-based live-line operation detection. Multi-scale selection, frequency-domain refinement, bidirectional interaction, and state-space context modeling are assigned to different feature stages according to their intended representational roles rather than applied as a uniform module stack.
- A code-consistent spatial–frequency enhancement pathway is implemented. Multi-scale responses from 3×3, 5×5, and 7×7 receptive fields are combined through channel-wise branch selection, while the real and imaginary Fourier components are independently refined and subsequently fused with the spatial representation through dual channel gates.
- The architecture is evaluated beyond aggregate mAP through repeated training, component and placement ablations, cross-architecture analysis, scale-stratified evaluation, class-level precision/recall/F1, comparison with representative detector families, an external VisDrone benchmark, and runtime profiling. Negative module interactions are retained in the analysis rather than interpreted as universally additive gains.

## 2. Related Work

### 2.1. UAV-Based Object Detection for Power Applications

UAV imagery combines wide-area coverage with flexible viewpoints and has become an important source of visual data for power inspection, component monitoring, and worksite observation. Public aerial benchmarks such as VisDrone expose the difficulty of small objects, dense scenes, occlusion, and unconstrained viewpoints [25]. More general detection architectures, including Faster R-CNN, YOLO-family detectors, and transformer-based detectors such as Deformable DETR, DINO, DAB-DETR, and D-FINE, provide a broad methodological basis for aerial object detection [26,27,28,29,30,31,32,33]. UAV-specific studies have subsequently introduced lightweight backbones, enhanced feature pyramids, attention-based representations, and DETR-style architectures to improve small-object detection under aerial viewpoints [3,4,6,13,34,35]. Recent UAV-oriented small-object detectors further emphasize lightweight feature preservation and selective fusion. YOLOSO improves a YOLOv11-based detector with a high-resolution P2 branch and redesigned feature-enhancement blocks [36]; DPFS-YOLO coordinates detail enhancement, foreground-guided selection, and semantic guidance to reduce missed and false detections in complex aerial scenes [37]; and EFSL-YOLO combines feature enhancement, gated convolution, and local multi-scale context fusion in a YOLOv13-based framework [38,39]. For lightweight small-UAV detection, Gao et al. replaced conventional downsampling with Haar-wavelet downsampling, redesigned the neck with a lightweight cross-scale context fusion module, and introduced a small-object-oriented detection head in an improved YOLOv11 architecture [40].

Power-oriented UAV detection has recently moved toward more application-specific targets. Recent work has investigated protective-equipment detection in 10 kV live-line operations and demonstrated the feasibility of UAV-compatible intelligent monitoring [22]. Other studies have targeted small power-line components in complex UAV imagery or focused directly on safety-distance estimation [23,24]. These studies demonstrate the value of application-specific visual perception, but their target vocabularies are generally centered on a particular PPE task, a specific infrastructure component, or a downstream safety variable. In contrast, the present dataset jointly represents the worker, key PPE, temporary insulation, and electrical structural references in a common scene vocabulary so that the detector can provide a broader object-level basis for downstream safety reasoning.

### 2.2. Multi-Scale Feature Fusion

Multi-scale feature fusion is a standard strategy for handling target-scale variation. FPN, PANet, and EfficientDet established widely used mechanisms for transferring semantic information across feature resolutions [7,8,9]. Subsequent work has explored extended pyramid structures, high-resolution representations, and attention-assisted fusion for small-object detection [10,11,12,13,14]. These approaches are effective, but many rely on fixed addition, concatenation, or sequential refinement. In live-line scenes, the appropriate receptive field may change substantially across object types and UAV viewpoints. A glove and a pole may occur in the same image while requiring very different contextual support. MSSF therefore introduces parallel receptive-field branches followed by content-conditioned weighting to retain multiple scale responses before fusion.

### 2.3. Frequency-Aware Representation and Context Modeling

Attention mechanisms such as SENet, CBAM, coordinate attention, and ECA-Net have demonstrated the benefit of feature reweighting for object discrimination [41,42,43,44]. Context-aware detection further shows that scene-level information can help disambiguate visually similar objects [15,45,46]. However, spatial attention alone does not explicitly expose frequency characteristics. Recent remote-sensing detectors have therefore explored frequency-aware representations from complementary perspectives. FANet uses multi-scale frequency enhancement and ROI-level attention for tiny-object detection [47], while HS-FPN introduces high-frequency perception and spatial-dependency modeling into the feature pyramid [48]. SET provides a different spectral view by suppressing background high-frequency responses and enhancing target saliency during training [49]. These studies motivate frequency-aware modeling for aerial imagery, but they do not specifically address the worker–PPE–insulation scene structure considered here. FreqAFU instead refines the real and imaginary Fourier components, reconstructs the frequency-domain response in the spatial domain, and combines it with the normalized spatial feature through separate learned channel gates. This formulation retains an explicit spatial pathway while allowing the frequency branch to contribute adaptively.

Global contextual interaction is also important when the worker, temporary insulation, and electrical structures are spatially separated. Transformer architectures can model long-range dependencies, but self-attention has quadratic complexity with respect to sequence length [18]. Structured state-space models, including Mamba, provide an alternative mechanism for long-context modeling with efficient scan-based computation [19,20,21]. The present framework combines Mamba with bidirectional cross-scale interaction so that contextual information is propagated together with feature-pyramid information. Recent remote-sensing work also highlights the value of structure-preserving and cross-modal feature modeling under complex visual conditions [50,51,52]. The relevance of these studies to the present work is methodological rather than task-equivalent: they support explicit treatment of structural information, while the proposed detector remains a single-image RGB object detector for live-line scenes.

### 2.4. Targeted Role and Differentiation of the Proposed Components

The methodological contribution of the framework lies at the architecture level. The elementary operations used by MSSF, FreqAFU, BiNet, and the Mamba context blocks are not presented as new primitives. Instead, the design assigns these operations to feature stages where they address different representation requirements observed in live-line UAV imagery. MSSF performs input-conditioned receptive-field selection during feature extraction. FreqAFU introduces a gated frequency-domain pathway while retaining a spatial feature stream. BiNet provides reciprocal cross-scale information exchange, and Mamba blocks are applied to compact semantic representations rather than to the highest-resolution maps. This organization is intended to avoid treating heterogeneous enhancement operations as interchangeable plug-ins. The positioning relative to representative related mechanisms is summarized in Table 1.

The resulting contribution is therefore the task-oriented coordination and placement of heterogeneous feature transformations within a common detector. This distinction is important for interpreting the ablations: the experiments are used to identify effective configurations and empirical boundaries, not to claim that every component is beneficial on every host architecture.

## 3. Materials and Methods

### 3.1. Safety-Oriented Problem Formulation

Let an image captured by a UAV be denoted by *I*. The objective is to predict a set of object detections

(1) $$D\left(I\right)={\left\{\left(b_{i},c_{i},s_{i}\right)\right\}}_{i=1}^{N},$$

where $b_{i}$ is the bounding box, $c_{i}$ is the category, and $s_{i}$ is the confidence score. The category set is partitioned into four semantic groups:

(2) $$C=C_{W}\cup C_{P}\cup C_{I}\cup C_{E},$$

where $C_{W}=\left\{\mathrm{person}\right\}$, $C_{P}=\left\{\mathrm{helmet},\;\mathrm{insulated}\;\mathrm{clothing},\;\mathrm{insulated}\;\mathrm{gloves}\right\}$, $C_{I}=\left\{\mathrm{insulated}\;\mathrm{cover},\;\mathrm{insulated}\;\mathrm{blanket}\right\}$, and $C_{E}=\left\{\mathrm{telegraph}\;\mathrm{pole},\;\mathrm{cross}\;\mathrm{arm}\right\}$. This semantic grouping defines the safety-relevant label space but does not alter the standard object-detection objective: no safety-compliance term is added to the detection loss. The detector therefore does not directly determine whether an operation complies with electrical safety regulations; instead, D(I) provides object-level visual evidence for downstream safety assessment.

### 3.2. Overall Framework

**Figure 1:**
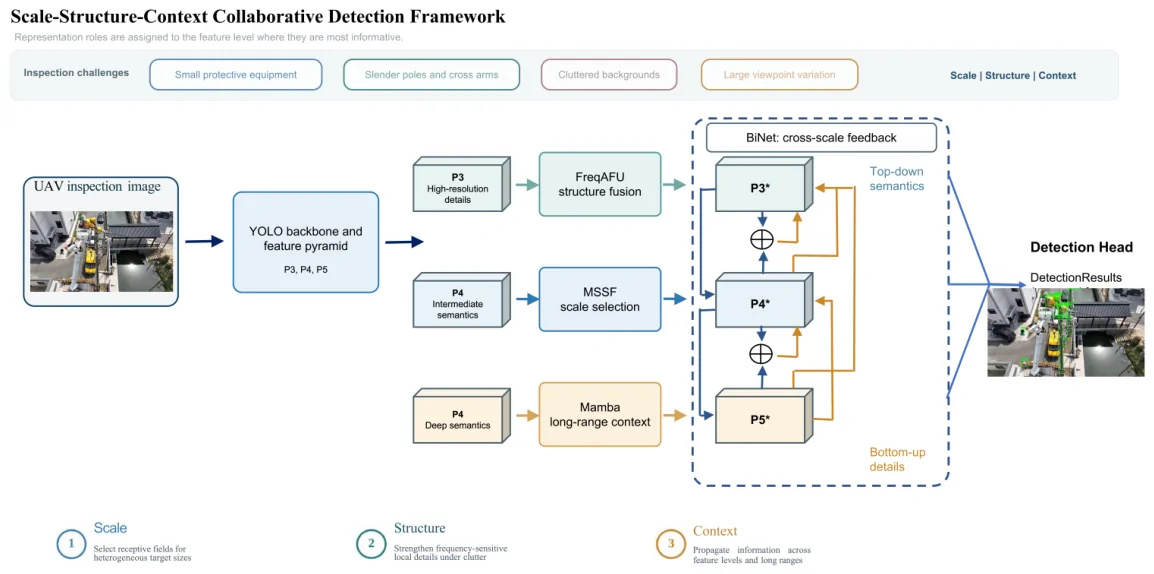
Overview of the proposed safety-oriented UAV detection framework. The detector extracts worker, PPE, temporary-insulation, and electrical-structure cues that can support downstream live-line safety assessment. MSSF, FreqAFU, and Mamba-BiNet address scale variation, structural/background interference, and long-range contextual interaction, respectively.

Figure 1 illustrates the proposed framework. A YOLO-style backbone extracts a hierarchical feature pyramid $\left\{P_{3},P_{4},P_{5}\right\}$. MSSF is inserted into selected feature extraction stages to adapt the effective receptive field. FreqAFU refines high-resolution representations by injecting frequency-domain structural residuals. The neck contains BiNet for reciprocal top-down and bottom-up information transfer, together with Mamba-based state-space blocks for long-range dependency modelling. The refined features are then delivered to the unchanged detection head.

The design follows a problem-to-mechanism mapping used as an architectural hypothesis rather than as a claim of mechanism-specific proof: MSSF is designed for scale variation, FreqAFU for structural refinement under background interference, and Mamba-BiNet for cross-scale and long-range contextual interaction. The experiments subsequently test the measurable consequences of these design choices; claims that are not directly isolated by an experiment are stated as design motivations rather than verified causal effects.

**Figure 2:**
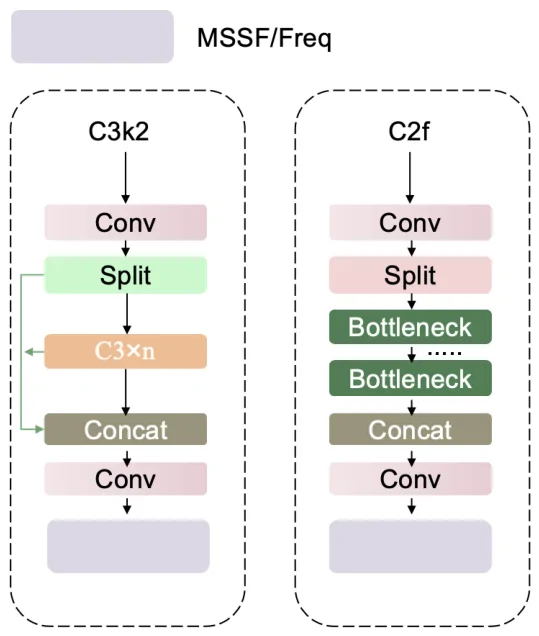
Block-level integration of the proposed feature enhancement. The original split-transform-concatenate topology is retained while selected building blocks receive multi-scale and frequency-awarerefinement. Black arrows indicate the main feature flow, green arrows indicate skip/aggregation paths, colors distinguish operation types, and the ellipsis denotes repeated bottleneck blocks.

Figure 2 shows the block-level integration used to preserve the original split–transform–concatenate topology while inserting the selected enhancement blocks.

### 3.3. Multi-Scale Selective Fusion (MSSF)

The MSSF block is implemented after the C2f feature transform and retains three parallel receptive-field branches with kernel sizes 3×3, 5×5, and 7×7. For an input feature map $X\in R^{C\times H\times W}$, the branch responses are

(3) $$X_{i}=B_{k_{i}}\left(X\right),\;k_{i}\in \left\{3,5,7\right\},$$

where each $B_{k_{i}}$ is a channel-preserving convolution followed by batch normalization and SiLU activation. The three responses are averaged and globally pooled:

(4) $$U=\frac{1}{3}\sum_{i=1}^{3}X_{i},\;s=\mathrm{GAP}\left(U\right)\in R^{C\times 1\times 1}.$$

The branch-weight generator uses two 1×1 projections with a ReLU nonlinearity. Its hidden width is

(5) $$d=\max\;\left(\left\lfloor \frac{C}{16}\right\rfloor ,8\right),$$

and the corresponding mapping is

(6) $$q=W_{2}\;\delta \left(W_{1}s\right),\;W_{1}:C\to d,\;W_{2}:d\to 3C,$$

where δ(·) denotes ReLU. After reshaping q to 3×C×1×1, Softmax is applied over the branch dimension:

(7) $$\alpha_{i,c}=\frac{\exp(q_{i,c})}{\sum_{j=1}^{3}\exp\left(q_{j,c}\right)},\;\sum_{i=1}^{3}\alpha_{i,c}=1.$$

The selected multi-scale response is then

(8) $$X_{\mathrm{MSSF}}=\sum_{i=1}^{3}\alpha_{i}⊙X_{i},\;X_{\mathrm{out}}=X+X_{\mathrm{MSSF}}.$$

Thus, the branch weights are channel-wise and input-dependent rather than fixed scalar coefficients.

**Figure 3:**
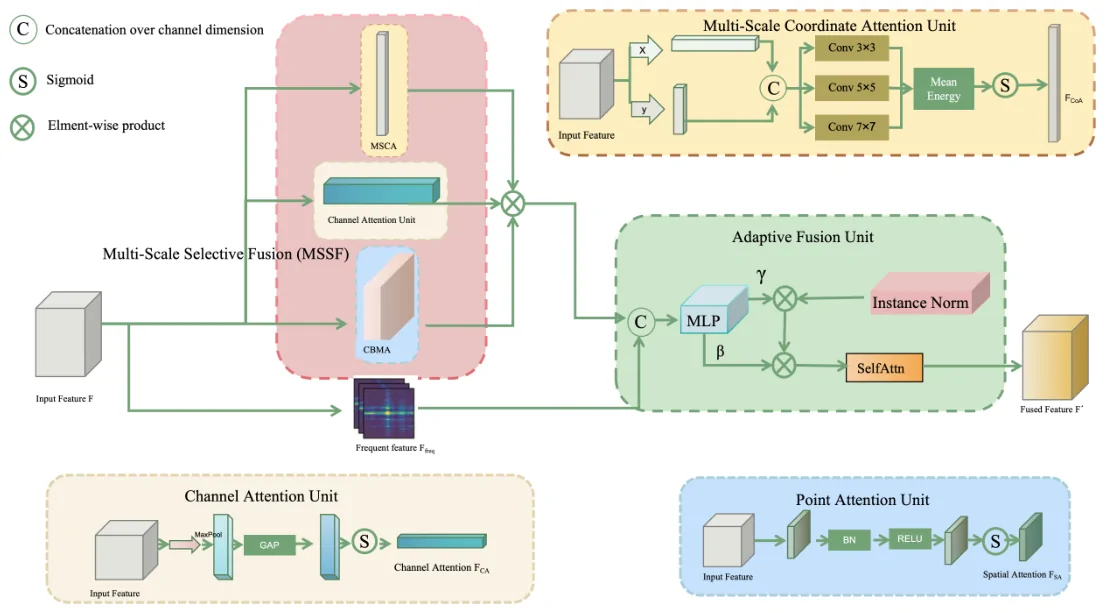
Implemented C2f-MSSF refinement. The MSSF core performs channel-wise selection among 3-by-3, 5-by-5, and 7-by-7 branches, followed by serial channel, point, and multi-scale coordinate attention.

In the implemented C2f-MSSF block, the MSSF output is followed by a serial attention refinement. Channel attention is generated from global average pooled features using a C→max(C/16,8)→C projection, point attention produces a single spatial weight map, and a coordinate-attention branch constructs normalized horizontal and vertical coordinate maps and processes them with 3×3, 5×5, and 7×7 convolutions. The coordinate responses are weighted by a Softmax computed from their mean responses and are applied after channel and point attention. Figure 3 summarizes this implemented sequence. The MSSF block is implemented after the C2f feature transform and retains three parallel receptive-field branches with kernel sizes 3×3, 5×5, and  7×7, as illustrated in Figure 2.

### 3.4. Frequency-Aware Adaptive Fusion (FreqAFU)

#### 3.4.1. Learnable Frequency-Domain Refinement

**Figure 4:**
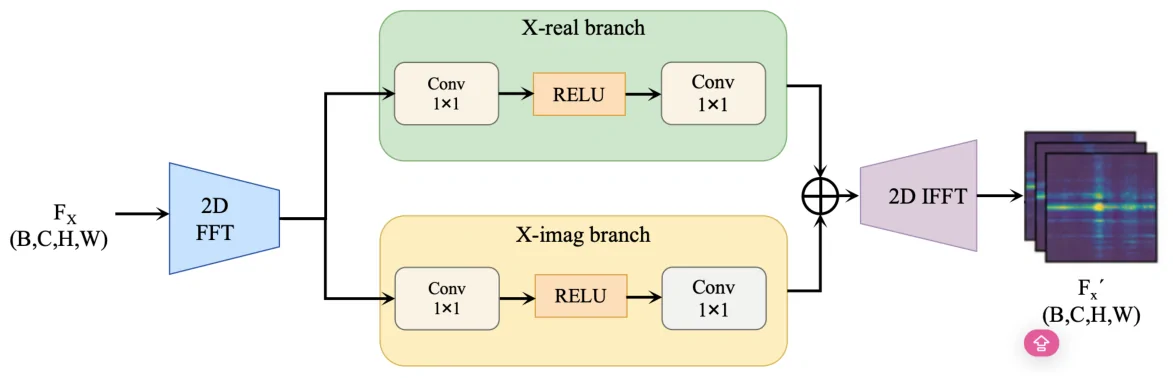
Implemented frequency branch in FreqAFU. A 2D FFT separates real and imaginary components; both are processed by shared 1-by-1 reduce–ReLU–restore projections and recombined before inverse FFT.

FreqAFU operates on the C2f feature before spatial-frequency fusion, as illustrated in Figure 4. Given $X\in R^{C\times H\times W}$, a two-dimensional Fourier transform is applied over the spatial dimensions:

(9) $$S=F_{2D}\left(X\right)=S_{r}+jS_{i}.$$

The implementation processes the real and imaginary components with the same pair of 1×1 projections. The intermediate frequency width is

(10) $$C_{f}=\max\;\left(8,\left\lfloor 0.5C\right\rfloor \right),$$

and the transformed components are

(11) $${\tilde{S}}_{r}=W_{2}^{f}\;\delta \left(W_{1}^{f}S_{r}\right),\;{\tilde{S}}_{i}=W_{2}^{f}\;\delta \left(W_{1}^{f}S_{i}\right),$$

where $W_{1}^{f}:C\to C_{f}$, $W_{2}^{f}:C_{f}\to C$, and δ(·) is ReLU. The two components are recombined and transformed back to the spatial domain:

(12) $$F_{f}=ℜ\;\left\{F_{2D}^{-1}\left({\tilde{S}}_{r}+j{\tilde{S}}_{i}\right)\right\}.$$

The FFT and inverse FFT are evaluated in floating-point precision and the real component of the inverse transform is returned to the detector feature stream. The operation therefore learns a frequency-domain refinement rather than applying a fixed high-frequency mask.

The implemented frequency branch is summarized in Figure 4.

#### 3.4.2. Adaptive Spatial–Frequency Fusion

The spatial path first applies MSSF and serial coordinate attention, yielding $F_{s}\in R^{C\times H\times W}$. The frequency branch produces $F_{f}$ from the same C2f feature. The two representations are concatenated and globally pooled:

(13) $$Z=\mathrm{Concat}\left(F_{s},F_{f}\right)\in R^{2C\times H\times W},\;z=\mathrm{GAP}\left(Z\right).$$

The adaptive fusion unit uses a two-layer 1×1 projection with hidden width

(14) $$C_{a}=\max\;\left(8,\left\lfloor \frac{C}{4}\right\rfloor \right),$$

followed by a split into two channel gates:

(15) $$\left[g_{s},g_{f}\right]=\psi \left(z\right),\;\psi :2C\to C_{a}\to 2C,$$

(16) $$\gamma =\sigma \left(g_{s}\right),\;\beta =\sigma \left(g_{f}\right).$$

Before fusion, the spatial feature is normalized by non-affine instance normalization. The gated representation is

(17) $$F_{g}=\gamma ⊙\mathrm{IN}\left(F_{s}\right)+\beta ⊙F_{f}.$$

Finally, the fused feature is refined by two 1×1 convolutions with an intermediate ReLU:

(18) $$F_{\mathrm{out}}=\rho \left(F_{g}\right),\;\rho :\;C\overset{1\times 1}{\to }C\overset{\mathrm{ReLU}}{\to }C\overset{1\times 1}{\to }C.$$

This formulation follows the implementation directly: the two gates independently regulate normalized spatial information and reconstructed frequency information, and the final 1×1 refinement performs channel mixing without introducing a separate self-attention matrix.

### 3.5. Bidirectional Feature Interaction Network (BiNet)

**Figure 5:**
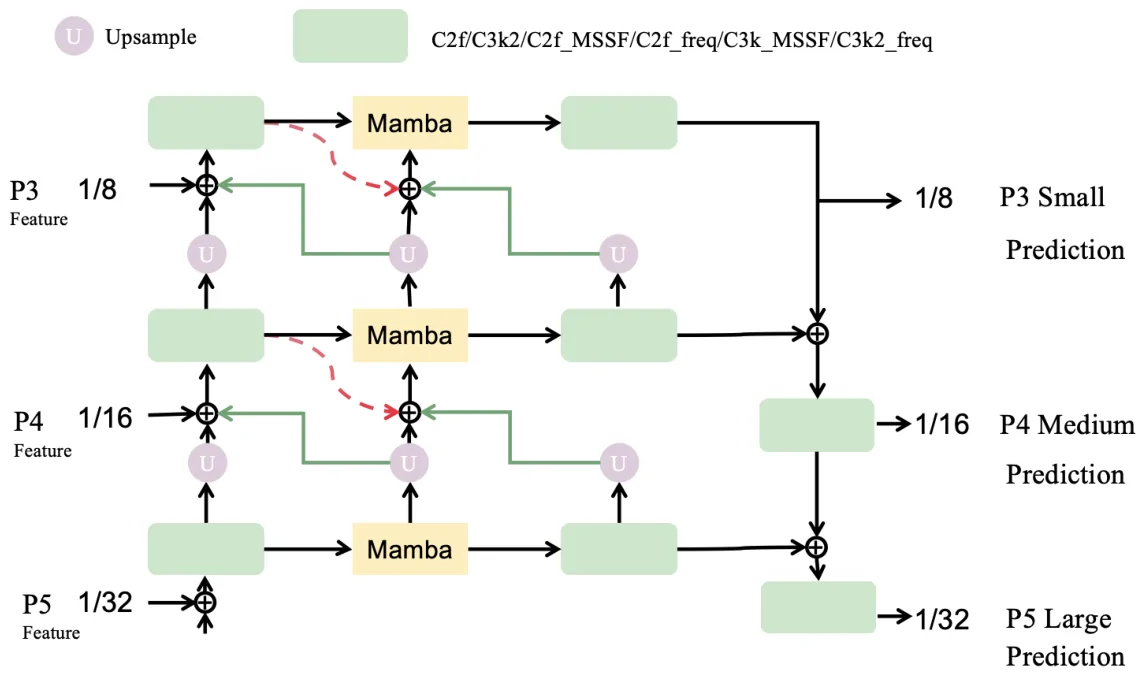
Bidirectional feature interaction with Mamba context blocks. Top-down paths propagate semantic information to higher-resolution features, while bottom-up paths return refined information to coarser levels before the three detection outputs. Black arrows denote the main feature-flow direction, green paths denote cross-scale feedback, U denotes upsampling, and circled plus signs denote feature fusion.

BiNet exchanges information across the $P_{3}$, $P_{4}$, and $P_{5}$ feature levels through top-down upsampling and bottom-up feedback, as shown in Figure 5. The conceptual updates can be written as

(19) $${\tilde{P}}_{l}=\Phi_{l}\;\left(P_{l},U\left({\tilde{P}}_{l+1}\right)\right),\;l\in \left\{4,3\right\},$$

(20) $${\hat{P}}_{l+1}=\Psi_{l+1}\;\left({\tilde{P}}_{l+1},D\left({\hat{P}}_{l}\right)\right),\;l\in \left\{3,4\right\},$$

where U(·) denotes spatial upsampling and D(·) denotes the implemented downsampling block. In the source implementation, the downsampling block consists of a depthwise 3×3 convolution with stride 2, batch normalization, a 1×1 channel projection, and a residual pointwise transform C→2C→C with SiLU activation.

For multi-level alignment, the gated fusion operator first projects the participating feature maps with 1×1 convolutions and resizes the auxiliary inputs to the spatial size of the reference feature using bilinear interpolation. Pairwise similarity maps are then obtained from channel-wise dot products, followed by sigmoid activation. These maps interpolate between the reference and aligned features before two pointwise refinement convolutions and a residual connection. This implementation makes the cross-scale exchange content-dependent while keeping the detection head unchanged. The term BiNet is used here to describe the bidirectional organization of these operations; bidirectional feature-pyramid fusion itself is not claimed as a new primitive. The bidirectional interaction pathway is illustrated in Figure 5.

### 3.6. Mamba-Based State-Space Context Modelling

**Figure 6:**
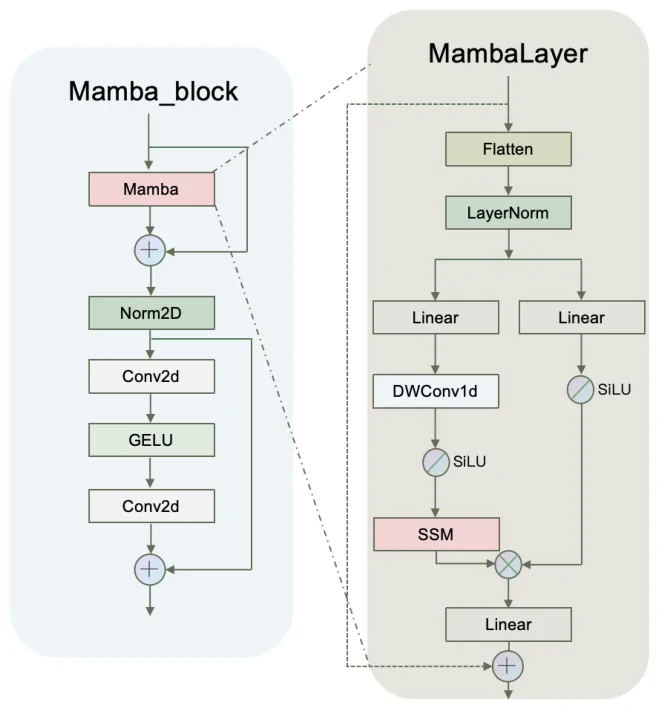
Mamba-based context block used in the detector. The feature map is flattened and normalized before selective state-space processing, followed by residual feature refinement. Arrows indicate feature flow, and residual/fusion symbols indicate element-wise feature addition.

The implemented Mamba wrapper operates on compact feature maps, as depicted in Figure 6. For an input $X\in R^{B\times C\times H\times W}$, the spatial dimensions are flattened to a sequence of length T=HW, producing $V\in R^{B\times T\times C}$. Layer normalization is applied before the Mamba operator:

(21) $$Y_{m}=\mathrm{Mamba}\left(\mathrm{LN}\left(V\right);d_{\mathrm{state}}=16,d_{\mathrm{conv}}=4,e=2\right).$$

The sequence output is reshaped back to B×C×H×W. In the wrapper used by the detector, this output is first combined with the input through a residual connection and batch normalization. A pointwise feed-forward transform then expands the channel width by a factor of two, applies GELU, projects it back to *C* channels, and adds a second residual connection:

(22) $$R=\mathrm{BN}(Y_{m}+X),$$

(23) $$Y=W_{2}^{m}\;\mathrm{GELU}\left(W_{1}^{m}R\right)+R,\;W_{1}^{m}:C\to 2C,\;W_{2}^{m}:2C\to C.$$

The Mamba operator internally uses a selective state-space scan with local convolution width four. The manuscript therefore treats the state-space equations as the internal sequence model rather than reproducing them as an additional hand-specified recurrence. Practical efficiency is evaluated from measured latency; the use of a scan-based state-space operator is not taken by itself as evidence of faster deployment. The internal organization of the context block is shown in Figure 6.

### 3.7. Feature-Collaboration Placement

The modules are placed according to a fixed design hypothesis: MSSF is applied at feature-extraction stages where several receptive fields can be retained before scale reduction; FreqAFU is applied to representations that preserve local structural detail; BiNet organizes the cross-scale interaction pathway; and Mamba blocks are inserted on comparatively compact semantic stages. MSSF placement is evaluated explicitly in Section 4.10. The positions of FreqAFU and Mamba are fixed design choices in the present study; because dedicated placement sweeps for those two modules are not reported, the manuscript does not claim that their selected positions are globally optimal.

## 4. Experimental Evaluation

### 4.1. Dataset and Safety-Oriented Category Design

The self-collected UAV dataset contains 8124 images and 118,119 annotated object instances. The eight categories are organized according to their downstream role in live-line safety perception. The person class identifies visible personnel who can serve as operator candidates for downstream association. Helmet, insulated clothing, and insulated gloves characterize essential PPE. Insulated cover and insulated blanket characterize temporary insulation measures. Telegraph pole and cross arm describe the surrounding electrical structure and provide spatial reference for subsequent geometric reasoning. The resulting label system provides the object categories needed for later worker–equipment association and spatial reasoning, but the detector itself does not assign a person instance to a specific operational role.

**Figure 7:**
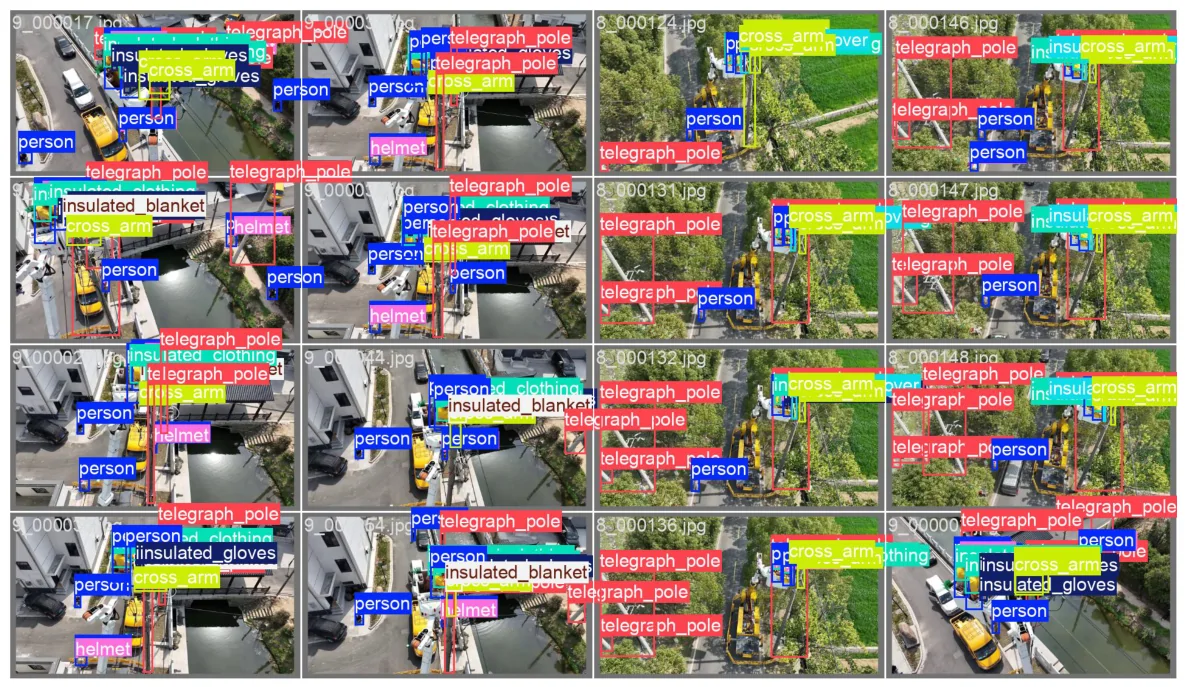
Representative annotated samples from the self-collected UAV live-line operation dataset. The examples contain workers, personal protective equipment, temporary insulating measures, and electrical structural references under different viewpoints, scales, and background conditions. Colored bounding boxes distinguish annotation classes, with the corresponding class name printed on each box; overlap occurs in densely annotated regions and does not indicate missing content.

Figure 7 presents representative annotated samples from the self-collected dataset. The examples contain visible workers, key protective equipment, temporary insulating measures, and electrical structural references under different viewpoints and target scales. Several samples also show cluttered vegetation, vehicles, shadows, and partial occlusion, which are representative of field acquisition conditions. The visual examples complement the quantitative dataset statistics by illustrating the variation in target scale, viewpoint, clutter, and occlusion present in the task.

**Figure 8:**
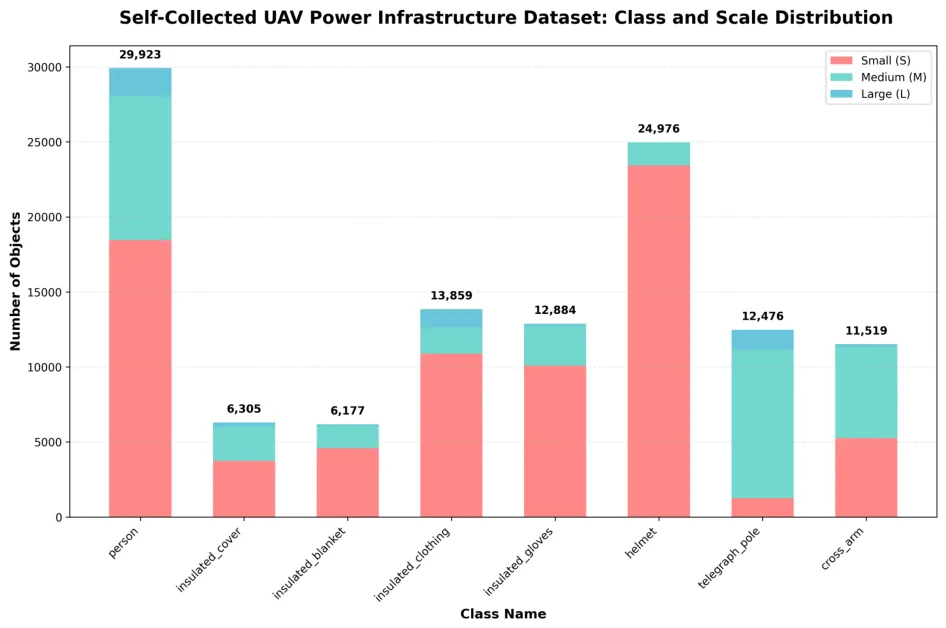
Semantic and scale composition of the self-collected UAV live-line operation dataset. The eight categories form four safety-oriented groups: worker, PPE, temporary insulation, and electrical structure.

**Figure 9:**
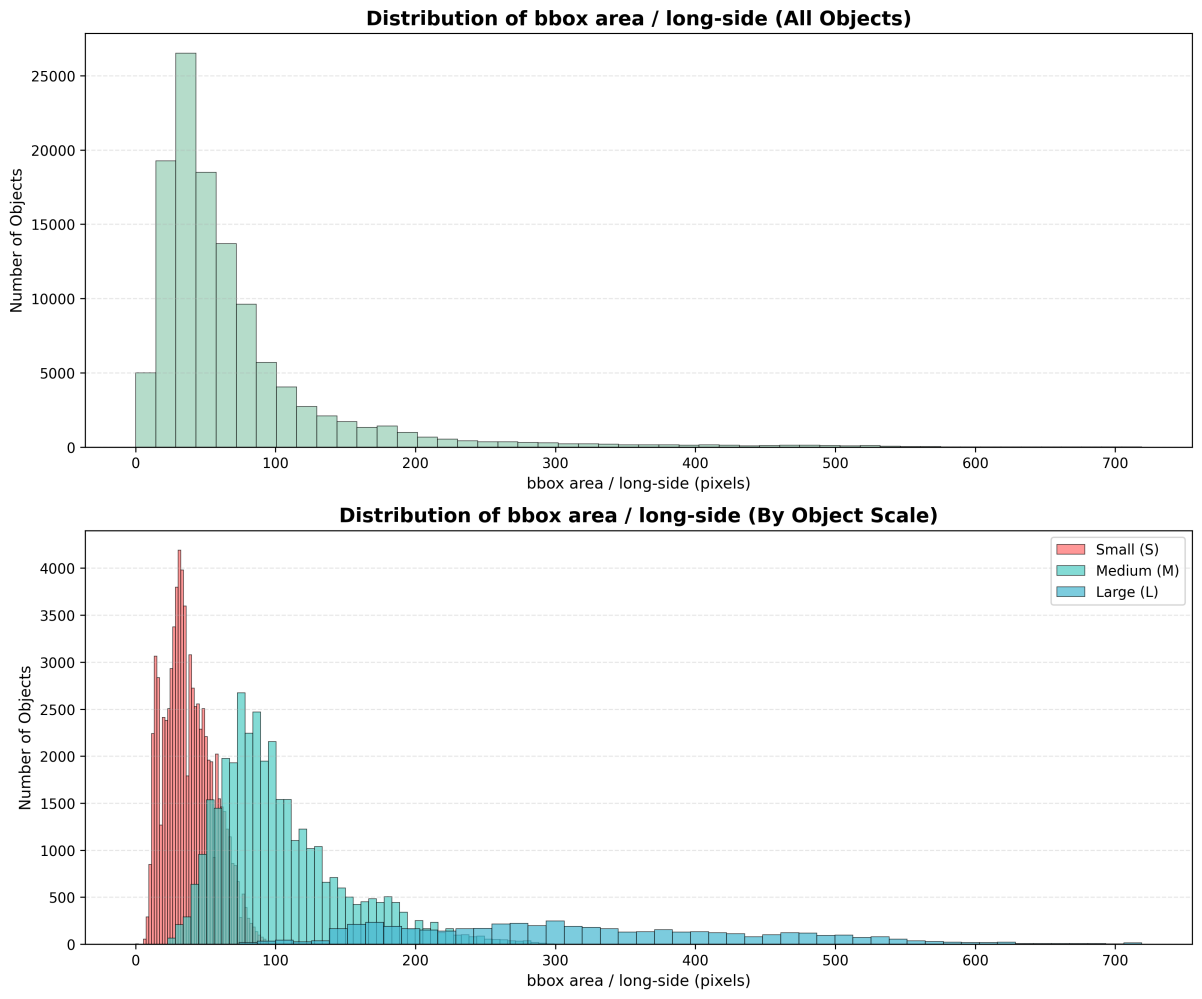
Distribution of object size measured by bounding-box long-side length. The presence of numerous small and slender objects motivates scale-aware feature extraction.

The category distribution is inherently imbalanced, as shown in Figure 8. Person and helmet have the largest instance counts, whereas insulated cover and insulated blanket are less frequent. In addition, protective equipment tends to occupy a smaller image area than poles and cross arms. The long-side distribution in Figure 9 therefore provides direct evidence that scale variation is an intrinsic property of the task rather than an incidental property of the benchmark.

The detector benchmark uses the complete 8124-image corpus, comprising 6499 training images and 1625 validation images. Partitioning is performed at the acquisition-session level rather than by random frame-level sampling: all frames from one acquisition session are assigned to only one split. This rule reduces leakage from temporally adjacent or near-duplicate UAV observations of the same work sequence. The validation split is kept fixed for all reported detector comparisons, ablations, and repeated runs. The live-line experiments therefore use a train/validation protocol and do not claim a separate held-out test set. Image–label file counts were verified as 6499 for training and 1625 for validation; the 118,119 total object instances and per-class instance counts are unchanged.

Table 2, Table 3 and Table 4 summarize the corpus statistics, per-class instance counts, and safety-oriented semantic organization, respectively.

### 4.2. Annotation Protocol and Quality Control

The dataset was annotated by approximately 20 annotators familiar with power-line inspection, UAV imagery, and object-detection tasks. The annotation workflow consisted of initial labeling followed by manual review. Occluded or partially visible objects were annotated when the visible region was sufficient to identify the object category. For partially occluded objects, the bounding box was drawn around the visible extent rather than extrapolating an unobserved boundary. Ambiguous regions that could not be assigned reliably to one of the eight categories were excluded. Formal inter-annotator agreement statistics were not recorded; this is treated as a limitation of the current dataset and is considered when interpreting the class-wise and scale-stratified results.

### 4.3. Implementation Details and Evaluation Protocol

Experiments are implemented with PyTorch 2.11.0 and Ultralytics 8.3.149 on NVIDIA GeForce RTX 4090 D GPUs. The PyTorch environment uses CUDA 13.0 and cuDNN 9.19. Unless otherwise stated, all YOLO-based models are trained for 150 epochs at an input resolution of 640 × 640 with a batch size of 4. The random seed is fixed to 0 for the standard single-run experiments. For the repeated-training reliability experiment, seeds 0, 1, and 2 are used while keeping the data partition fixed.

Optimization uses the Ultralytics MuSGD variant with an initial learning rate of 0.01, momentum of 0.9, and weight decay of 0.0005. The training warm-up follows the default Ultralytics setting of 3 epochs. The loss weights for bounding-box regression, classification, and distribution focal loss (DFL) are 7.5, 0.5, and 1.5, respectively. Mosaic augmentation, horizontal flipping, and HSV perturbation are applied during training. Random erasing is enabled with a probability of 0.4.

The primary evaluation metrics are mAP_50_ and mAP_50-95_. Class-wise AP_50-95_, precision, recall, and F1 are additionally reported for the eight safety-oriented categories. Parameter count, THOP-estimated GFLOPs, latency, and FPS are used to characterize model complexity and inference efficiency. All GFLOPs values reported in the following tables are profiler-based estimates obtained with THOP 2.0.19 at 640 × 640 input resolution. Functional FFT/IFFT operations implemented through torch.fft are not included in the THOP count; the reported values are therefore not interpreted as exhaustive arithmetic-operation counts.

For inference-speed evaluation, each model is first warmed up for 50 forward passes, followed by 500 timed inference runs at batch size 1 and 640 × 640 input resolution. CUDA synchronization is applied immediately before and after timing to reduce error caused by asynchronous GPU execution. The reported latency is the arithmetic mean per-image inference time under the same hardware and precision setting, and FPS is calculated from the reciprocal of the mean latency. Data loading, CPU preprocessing, and post-processing are excluded from the detector-side timing.

### 4.4. Reproducibility and Model Availability

The source code and the self-collected UAV dataset used in this study will be made publicly available at https://github.com/gfjgfjgfj/UAV (accessed on 13 October 2026). The repository will provide the dataset organization and annotations together with the model YAML files, module source definitions, training hyperparameters, random seeds, training commands, evaluation commands, and environment information used for the reported experiments. The standard single-run experiments use random seed 0. The repeated-training reliability experiment uses seeds 0, 1, and 2 with the same data partition fixed across runs.

The implementation details of MSSF, FreqAFU, BiNet, and the Mamba context blocks are specified through both the method description and the corresponding configuration/source files. MSSF is defined by its parallel receptive-field branches, content-conditioned branch weighting, and residual fusion. FreqAFU is defined by the FFT-based real/imaginary refinement, inverse transform, channel-wise dual gating, and spatial-frequency fusion. BiNet is defined by its top-down semantic propagation and bottom-up localization-feedback paths. The Mamba blocks are placed on compact semantic feature maps within the bidirectional interaction pathway rather than on high-resolution feature maps. Together with the model configuration and module source files, these details are intended to make the architecture reconstructable without requiring access to the raw imagery; the equations alone are not presented as a complete implementation specification.

The public repository will include the dataset, annotation schema, benchmark partition description, model configurations, and executable training and evaluation scripts needed to reproduce the reported experiments. Repository contents will be organized to correspond directly to the experimental settings described in this paper.

### 4.5. Baseline Performance

Table 5 reports the baseline results for Ultralytics YOLOv8 [28] and YOLO11 [29]. The model families exhibit the expected capacity–accuracy trend and provide a consistent reference for evaluating the proposed feature enhancements.

### 4.6. Dataset Partition Verification

Table 6 summarizes the full train/validation partition used in the experiments. Models are optimized using the 6499-image training split, while all live-line detector results reported in this section are evaluated on the same fixed 1625-image validation split. The validation split is not used for gradient-based optimization. Because no independent live-line test split is configured, all performance claims in this paper are explicitly reported as validation-set results.

### 4.7. Statistical Reliability

The main baseline and enhanced YOLOv8n configurations are independently trained three times with fixed seeds. Table 7 reports mean ± standard deviation on the fixed 1625-image validation set. The variation is small relative to the observed improvement, indicating that the reported gain is not attributable to a single stochastic training run.

### 4.8. Overall Detection Results

Table 8 summarizes class-wise AP_50-95_ and aggregate detection performance for the enhanced configurations. The largest relative gain is observed on YOLOv8n, where mAP_50-95_ increases from 0.558 to 0.635. The improvement becomes smaller as the host detector capacity increases, indicating that the benefit of additional feature collaboration is capacity-dependent.

### 4.9. Component Ablation

A component ablation is first performed on YOLOv8n, which is the primary lightweight host architecture used for the main claim. The same data partition and training protocol are retained across the ablation rows. Table 9 shows that the gain is interaction-dependent rather than a simple sum of isolated module effects. Adding Mamba+BiNet increases mAP_50-95_ from 0.558 to 0.631, indicating that long-range context is most effective when coupled with bidirectional cross-scale interaction. Adding FreqAFU gives a small further improvement in localization quality, and the full configuration with MSSF achieves the best overall result of $0.949\;{\mathrm{mAP}}_{50}$ and 0.635 mAP_50-95_.

**Figure 10:**
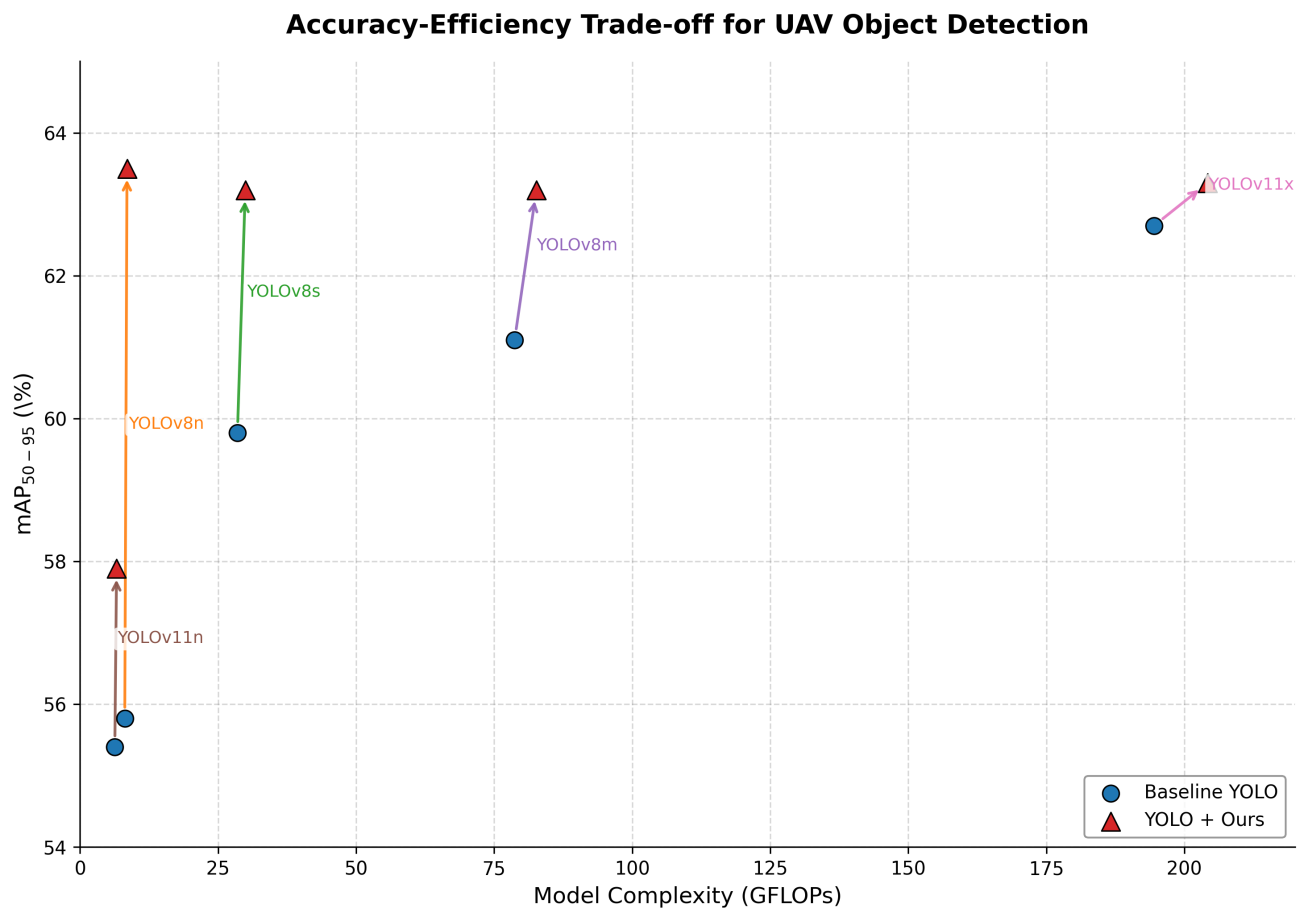
Accuracy–efficiency comparison of selected YOLO baselines and enhanced configurations on the live-line UAV benchmark. Circles denote baseline YOLO models, triangles denote the corresponding enhanced configurations, and arrows connect each baseline with its enhanced counterpart.

Table 9 summarizes this unified-backbone ablation, while Figure 10 visualizes the accuracy–efficiency relationship of the selected YOLO baselines and enhanced configurations.

The YOLOv11n experiment is retained as a cross-architecture interaction analysis rather than being used as evidence that every component must be beneficial for every host. As shown in Table 10, Mamba+BiNet improves mAP_50-95_ from 0.554 to 0.579, while the additional FreqAFU branch reduces it to 0.575. After MSSF is incorporated, however, the complete YOLOv11n + proposed configuration (Mamba+BiNet+FreqAFU+MSSF) reaches $0.920\;{\mathrm{mAP}}_{50}$ and 0.585 mAP_50-95_, which is the best result among the tested YOLOv11n variants. This sequence shows that the contribution of FreqAFU is configuration dependent: it is not beneficial when added to Mamba+BiNet alone, but the final fused configuration recovers the loss and achieves the highest overall accuracy after multi-scale selective fusion is introduced. Because the current experiments do not isolate the internal feature statistics responsible for this interaction, no stronger causal explanation is inferred from the ablation alone.

The YOLOv11n ablation therefore does not support a purely additive interpretation of the components. The intermediate decrease after adding FreqAFU and the subsequent improvement after adding MSSF indicate that the complete configuration should be evaluated as an interacting architecture rather than as the sum of independent plug-in gains. The main contribution remains the task-oriented allocation of different feature transformations to different stages, with the useful configuration dependent on the host architecture.

### 4.10. Cross-Architecture Comparison

Table 11 compares the enhanced configurations across the YOLOv8 and YOLOv11 families. The results show that the magnitude of the improvement depends on the host architecture, supporting the use of cross-architecture evaluation rather than assuming a fixed gain across detector families.

### 4.11. Comparison with Representative Contemporary Detectors

To broaden the comparison beyond the YOLO family, representative detectors from two-stage convolutional, transformer-based, UAV-oriented, lightweight anti-UAV, and state-space detection directions are re-evaluated on the same live-line benchmark. All results in Table 12 are obtained using the fixed 6499/1625 train/validation partition and the unified evaluation protocol adopted in this study. Faster R-CNN provides a two-stage reference [26], RT-DETR-R18 represents a real-time DETR-family detector [54], UAV-DETR represents a UAV-specific transformer detector [34], the lightweight anti-UAV model provides a compact YOLOv11-based small-target reference [40], and Mamba-YOLO represents state-space-based detection [21].

The updated comparison shows that the proposed model achieves the highest accuracy among the evaluated detectors, reaching $0.949\;{\mathrm{mAP}}_{50}$ and 0.635 mAP_50-95_. YOLO26n provides a recent compact baseline, reaching $0.929\;{\mathrm{mAP}}_{50}$ and 0.586 mAP_50-95_ with 2.38 M parameters and 5.3 GFLOPs. Among the external compact references, Lightweight Anti-UAV uses 3.4 M parameters and 15.2 GFLOPs while reaching 0.602 mAP_50-95_, illustrating a strong lightweight operating point. Mamba-YOLO reaches 0.600 mAP_50-95_ with 12.86 M parameters, while UAV-DETR, RT-DETR, and RT-DETR-X provide transformer-based reference points at higher computational cost. Compared with larger vanilla YOLO models, the proposed configuration also exceeds YOLOv8x (0.624) and YOLOv11x (0.627) in mAP_50-95_ while using fewer parameters than either large-scale baseline. These results show that the proposed configuration remains competitive across the evaluated detector families without implying universal superiority.

### 4.12. External UAV Benchmark: VisDrone

VisDrone2019-DET is used as an external UAV benchmark because its label space and scene distribution differ substantially from the live-line safety task [25]. The benchmark contains 6471 training images, 548 validation images, and 1610 test-dev images across ten object classes. The official train split is used for optimization, the validation split is used for model selection, and the test-dev split is used for final evaluation. No live-line images are mixed into the VisDrone training data.

The controlled comparison includes YOLOv8n, YOLOv11n, the selected YOLOv8n- and YOLOv11n-based enhanced configurations, Faster R-CNN with a ResNet-50 backbone, RT-DETR, RT-DETR-X, and Mamba-YOLO. Baseline YOLO models are initialized from the corresponding COCO-pretrained checkpoints, while the enhanced models use the same host-detector initialization and randomly initialized added modules. All models in the controlled group are fine-tuned on VisDrone at 640 × 640 resolution for 150 epochs with batch size 4 and the same evaluation metrics. Parameter count and GFLOPs are reported where they are available from the corresponding implementation. For additional context, Table 13 also reports published VisDrone results for YOLOSO [36], DPFS-YOLO [37], and EFSL-YOLO [38]. These literature values are kept in a separate group because their training schedules, optimization settings, and evaluation details differ from the unified protocol used in this study.

The two result groups in Table 13 serve different purposes. Within the controlled group, the YOLOv8n-based proposed configuration reaches $0.441\;{\mathrm{mAP}}_{50}$ and 0.272 mAP_50-95_, while Mamba-YOLO is the strongest non-proposed reference under the same experimental protocol. The literature-reported rows provide contemporary context only: YOLOSO reports 0.220 mAP_50-95_, DPFS-YOLO 0.228, and EFSL-YOLO 0.243 on VisDrone2019-DET [36,37,38]. Because these studies use different training schedules and implementation settings, the numerical differences are not treated as strict head-to-head gains. The external benchmark is therefore used to assess transfer behavior and to position the proposed detector relative to recent UAV small-object detection research, rather than to make a leaderboard claim.

### 4.13. Safety-Oriented Class-Level Evaluation

Because the intended application is safety-oriented perception, aggregate mAP is not sufficient by itself. False negatives are especially important for PPE categories because a missed helmet, insulated glove, or protective garment would remove the visual evidence needed by a downstream safety-assessment module. Therefore, class-level precision, recall, and F1 are reported for the selected YOLOv8n configuration. As shown in Table 14, the detector maintains high precision and recall for person, helmet, telegraph pole, and cross arm, but the smallest PPE categories remain more difficult. Insulated gloves have the lowest recall among the PPE-related classes, indicating that missed detections are still the primary safety-oriented weakness of the current perception model.

The main false-negative cases occur when gloves occupy only a few pixels, are partially hidden by hands, tools, or the bucket edge, or appear against cluttered backgrounds with similar colors. Helmet misses are less frequent but still occur under strong viewpoint changes, motion blur, or partial occlusion by the worker’s body or surrounding equipment. Insulated covers and blankets can also be confused with background materials when their visible boundaries are weak. These cases illustrate the remaining difficulty of small, occluded, and weak-boundary PPE targets, but they also delimit the current scope: the detector provides object-level evidence and does not by itself infer whether a particular worker is compliant with a site-specific electrical safety rule. In a field safety system, low-confidence or missing PPE detections should therefore be treated as cues for downstream temporal verification or manual review rather than as final compliance decisions.

### 4.14. Mechanism-Specific Validation

To connect the architectural claims with measurable image characteristics, the fixed validation set is additionally stratified by bounding-box long-side length measured after resizing to the 640 × 640 evaluation resolution. The diagnostic bins use long-side thresholds of ≤32 pixels (small), 32–96 pixels (medium), and >96 pixels (large). These bins are used for within-study diagnostic comparison and are not claimed to be identical to the area-based COCO small/medium/large definition. Table 15 compares the baseline with the principal module configurations.

The largest relative gain is observed for small objects. This result provides direct support for the scale-related motivation of the multi-scale branch, because MSSF is the component explicitly designed to combine different receptive-field responses. The remaining configurations also improve one or more scale groups, but the present scale stratification does not isolate the specific mechanisms of FreqAFU, BiNet, or Mamba. Accordingly, these results are interpreted as configuration-level evidence rather than as causal proof of each individual component.

This scale-stratified analysis provides direct evidence for the scale-related mechanism claim, but it does not fully isolate all scene factors discussed in the motivation. Clutter level, occlusion level, and worker–equipment spatial separation are not explicitly annotated as independent attributes in the current dataset. Reporting AP under those factors would therefore require additional attribute labeling or a dedicated diagnostic subset. We consequently restrict the mechanism-specific claim in this paper to the measured scale-stratified evidence and treat clutter-, occlusion-, and spatial-separation-stratified evaluation as future work.

## 5. Discussion

### 5.1. Safety Relevance of the Eight-Class Object Vocabulary

The class design should not be interpreted as a reduced version of a generic object-detection benchmark. The categories are selected according to the information needed to interpret a live-line work scene. The person class provides candidate worker instances for downstream association. PPE categories identify key protective wear. Insulated cover and blanket indicate temporary insulating measures. Pole and cross arm define the electrical structure relative to which worker and protection objects can be located. This semantic structure allows the detector to serve as an upstream perception layer for a safety-monitoring system.

Object detection alone is insufficient to verify compliance with electrical safety regulations. A downstream module must associate PPE and temporary insulation with individual workers or energized structures, estimate relevant spatial relationships, and apply explicit geometric or rule-based constraints. The proposed detector therefore supplies the object-level evidence required by a subsequent reasoning layer rather than replacing that layer.

### 5.2. Architectural Interaction and Scope of the Enhancement Mechanisms

The ablation results indicate that the proposed components should not be interpreted as independent plug-in modules whose effects accumulate monotonically. On YOLOv8n, Mamba+BiNet provides the dominant improvement, FreqAFU produces only a small additional change in mAP_50-95_, and the complete configuration reaches the highest accuracy after MSSF is included. On YOLOv11n, adding FreqAFU to Mamba+BiNet decreases mAP_50-95_ relative to Mamba+BiNet alone. This result establishes an empirical boundary of the frequency-domain branch rather than evidence of universally complementary behavior.

The scale-stratified experiment provides the clearest direct evidence for the role of multi-scale processing. The largest improvement is observed for small targets, which is consistent with the motivation for retaining and selecting among responses from multiple receptive fields. However, the current dataset does not separately annotate background clutter, occlusion severity, or worker–equipment spatial separation. Consequently, the roles assigned to frequency-domain refinement, bidirectional interaction, and state-space context modeling should be interpreted as design motivations supported by aggregate and ablation behavior rather than as individually isolated causal mechanisms.

This distinction is important for interpreting the architecture. The framework does not assume that Fourier processing, bidirectional fusion, or state-space modeling is intrinsically superior to alternative operators. Module placement and interaction are therefore treated as part of architecture design rather than as fixed transferable rules.

The MSSF placement study further supports a cost-aware design perspective. The serial configuration produces the highest score but adds 16.00 M parameters and 35.8 THOP-estimated GFLOPs, whereas parallel or single-level variants provide lower-cost alternatives. Runtime is reported separately because the profiler does not count all custom operations, including the functional FFT/IFFT calls used by FreqAFU. The deployment results should therefore be interpreted from measured latency together with parameter count and profiler-based complexity, rather than from GFLOPs alone.

### 5.3. Relationship to Recent Power-Safety Detection Research

Recent work has already demonstrated that UAV-compatible object detection can support live-line protective-equipment monitoring and edge deployment [22]. Other power-oriented studies focus on safety-distance estimation or specialized PPE datasets [24]. The contribution of the present work is different in emphasis: rather than solving the final safety decision directly, it constructs a broader object-level scene representation containing the worker, PPE, temporary insulation, and electrical structural references. This representation can be coupled with downstream geometric or rule-based safety reasoning in future work.

### 5.4. Deployment Considerations and Future Extensions

The present evaluation focuses on image-level detection. A complete field-deployment pipeline should additionally consider temporal consistency across UAV video, end-to-end latency, memory footprint, and energy consumption on the target edge platform. Safety-state inference also requires explicit association of workers with PPE and temporary insulation, geometric relations to energized structures, and site-specific operational rules. These requirements define the next stage of system development beyond the object-level perception model evaluated here.

A natural extension is to convert the detected objects into a structured scene representation. Workers, PPE, insulating materials, poles, and cross arms can be represented as nodes, with spatial relations forming edges. Geometric distances and rule-based constraints can then be applied to infer safety-related states. This two-stage formulation separates generic visual perception from site-specific safety rules and provides a practical route toward automated UAV-assisted safety assessment.

## 6. Conclusions

This study develops a safety-oriented UAV object detection framework for live-line power operations. The detector is designed to perceive visible personnel, key PPE, temporary insulating measures, and electrical structures that can serve as object-level evidence for subsequent safety analysis. The self-collected dataset contains 8124 annotated images and 118,119 object instances across eight semantically organized categories and is evaluated under a fixed acquisition-session-level train/validation protocol.

The architecture coordinates multi-scale selection, frequency-domain refinement, bidirectional feature interaction, and state-space context modeling at different feature stages. The contribution is therefore an architecture-level organization of heterogeneous feature transformations rather than a claim that the underlying operators are individually new. On the fixed live-line benchmark, the complete YOLOv8n-based configuration improves mAP_50-95_ from 0.558 to 0.635 and ${\mathrm{mAP}}_{50}$ from 0.900 to 0.949. Repeated training, placement analysis, cross-architecture ablation, class-level metrics, scale-stratified evaluation, representative-detector comparison, the VisDrone benchmark, and runtime profiling are used to characterize both the gains and the limits of the design.

The experiments also show that the components are not universally additive. In particular, FreqAFU provides a weaker interaction on YOLOv11n than on YOLOv8n, and the scale-stratified analysis directly supports the multi-scale motivation more strongly than it supports causal claims for the other components. These findings favor a configuration-dependent interpretation in which feature transformations are selected jointly with the host architecture and computational budget.

The detector is positioned as the visual perception layer of a broader safety-monitoring system. It does not itself perform worker–PPE association, safety-distance estimation, behavioral-state inference, or regulatory compliance decisions. Future work should therefore evaluate temporal consistency in UAV video, explicit relational reasoning, and end-to-end deployment on representative edge hardware.

## Figures and Tables

**Table 1:** Technical positioning of the proposed components relative to representative related mechanisms.

Component	Representative Related Direction	Common Limitation in the Present Task	Targeted Distinction in This Work	Evidence
MSSF	FPN/PAN/BiFPN-style multi-scale fusion and multi-branch receptive-field aggregation	Fixed or predefined cross-scale aggregation does not explicitly select among receptive fields at the feature-extraction stage.	Parallel 3×3, 5×5, and 7×7 responses are retained and combined through input-conditioned channel-wise branch weights before residual fusion.	the placement experiment and scale-stratified analysis
FreqAFU	FANet, HS-FPN, SET and related frequency-aware tiny-object detectors	Frequency-domain processing may be redundant or detrimental for some host feature distributions.	Real and imaginary Fourier components are independently refined, reconstructed in the spatial domain, and combined with the normalized spatial feature through separate learned channel gates.	the unified-backbone and cross-architecture ablation analyses
BiNet	PANet/BiFPN and bidirectional feature-pyramid interaction	One-way propagation may limit reciprocal exchange between localization-oriented and semantic feature levels.	A top-down and bottom-up interaction path is used to exchange information in both directions before detection-head prediction.	the unified-backbone and cross-architecture ablation analyses
Mamba context block	Mamba-based detection and state-space modelling	Direct long-sequence modelling on high-resolution maps can increase computational burden.	State-space processing is restricted to compact semantic feature maps and used together with the bidirectional interaction pathway.	the unified-backbone ablation and cross-architecture comparison
Complete framework	Direct stacking of heterogeneous enhancement modules	The effect of a component can depend on the host backbone and feature topology.	The architecture is treated as a configuration-dependent feature-collaboration design, and negative cross-architecture interactions are reported explicitly.	the unified-backbone and cross-architecture ablation analyses; the complexity analysis

**Table 2:** Corpus statistics of the self-collected UAV live-line operation dataset.

Statistic	Value
Annotated images	8124
Object instances	118,119
Object categories	8
Training images	6499
Validation images	1625

**Table 3:** Object-instance counts by category in the complete corpus.

Category	Instances
person	29,923
insulated cover	6305
insulated blanket	6177
insulated clothing	13,859
insulated gloves	12,884
helmet	24,976
telegraph pole	12,476
cross arm	11,519

**Table 4:** Safety-oriented semantic organization of the self-collected dataset.

Semantic Group	Classes	Perception Role
Worker	person	Identifies visible personnel as candidate worker instances and provides reference objects for subsequent worker-centered association and analysis.
PPE	helmet; insulated clothing; insulated gloves	Characterizes the key personal protective equipment required during live-line operations.
Temporary insulation	insulated cover; insulated blanket	Represents temporary insulating measures used to isolate or protect potentially energized components.
Electrical structure	telegraph pole; cross arm	Provides structural and spatial references for interpreting the worker’s operating environment and subsequent safety relationships.

**Table 5:** Baseline performance of YOLOv8 and YOLOv11 on the self-collected dataset.

Model	Params (M)	GFLOPs	mAP_50_	mAP_50-95_
YOLOv11n	2.59	6.5	0.896	0.554
YOLOv11s	9.42	21.3	0.926	0.593
YOLOv11m	20.04	67.7	0.936	0.613
YOLOv11l	25.29	86.6	0.941	0.620
YOLOv11x	56.84	194.5	0.945	0.627
YOLO26n [53]	2.38	5.3	0.929	0.586
YOLOv8n	3.01	8.1	0.900	0.558
YOLOv8s	11.13	28.5	0.929	0.598
YOLOv8m	25.84	78.7	0.934	0.611
YOLOv8l	43.61	164.8	0.938	0.619
YOLOv8x	68.13	257.4	0.942	0.624

**Table 6:** Full-dataset partition used for model training and evaluation.

Model	Train	Validation	mAP_50_	mAP_50-95_
YOLOv8n	6499	1625	0.900	0.558
YOLOv8n + proposed	6499	1625	0.949	0.635

**Table 7:** Three-run statistical results on the fixed 1625-image validation set.

Model	mAP_50_	mAP_50-95_
YOLOv8n	0.900±0.003	0.558±0.004
YOLOv8n + proposed	0.949±0.002	0.635±0.003

**Table 8:** Class-wise AP_50-95_ and aggregate detection performance of selected enhanced configurations on the self-collected dataset.

Model	Person	Ins_Cover	Ins_Blanket	Ins_Cloth	Ins_Gloves	Helmet	Pole	Cross_Arm	mAP_50_	mAP_50-95_
YOLOv8n + proposed	0.690	0.610	0.600	0.645	0.430	0.550	0.795	0.760	0.949	0.635
YOLOv8x + proposed	0.705	0.635	0.620	0.665	0.445	0.565	0.810	0.747	0.956	0.649
YOLOv11n + proposed	0.618	0.608	0.601	0.621	0.337	0.456	0.772	0.669	0.920	0.585
YOLOv11x + proposed	0.695	0.625	0.615	0.655	0.440	0.555	0.805	0.674	0.950	0.633

**Table 9:** YOLOv8n ablation of the proposed interaction modules. A check mark indicates that the corresponding component is enabled.

YOLOv8n	Mamba+BiNet	FreqAFU	MSSF	mAP_50_	mAP_50-95_
✓				0.900	0.558
✓	✓			0.947	0.631
✓	✓	✓		0.946	0.632
✓	✓	✓	✓	0.949	0.635

**Table 10:** Component interaction ablation on YOLOv11n, including the complete fused configuration. A check mark indicates that the corresponding component is enabled.

Mamba	BiNet	FreqAFU	MSSF	mAP_50_	mAP_50-95_
				0.896	0.554
		✓		0.898	0.555
✓				0.907	0.568
	✓			0.910	0.575
✓	✓			0.918	0.579
✓	✓	✓		0.912	0.575
✓	✓	✓	✓	0.920	0.585

**Table 11:** Cross-architecture comparison of baseline and selected enhanced configurations.

Detector	Baseline mAP_50-95_	Selected Configuration	Final mAP_50_	Final mAP_50-95_
YOLOv8n	0.558	Full feature collaboration	0.949	0.635
YOLOv8x	0.624	Full feature collaboration	0.956	0.649
YOLOv11n	0.554	Full feature collaboration	0.920	0.585
YOLOv11x	0.627	Full feature collaboration	0.950	0.633

**Table 12:** Comparison with representative detectors under the unified live-line benchmark.

Detector	Params (M)	GFLOPs	mAP_50_	mAP_50-95_
YOLOv11n	2.59	6.5	0.896	0.554
YOLOv11s	9.42	21.3	0.926	0.593
YOLOv11m	20.04	67.7	0.936	0.613
YOLOv11l	25.29	86.6	0.941	0.620
YOLOv11x	56.84	194.5	0.945	0.627
YOLOv8n	3.01	8.1	0.900	0.558
YOLOv8s	11.13	28.5	0.929	0.598
YOLOv8m	25.84	78.7	0.934	0.611
YOLOv8l	43.61	164.8	0.938	0.619
YOLOv8x	68.13	257.4	0.942	0.624
Faster R-CNN (ResNet-50) [26]	41.2	134.8	0.899	0.499
RT-DETR [54]	32.82	109.9	0.850	0.476
RT-DETR-X [54]	65.48	224.7	0.937	0.577
UAV-DETR [34]	22.6	66.1	0.895	0.550
Lightweight Anti-UAV [40]	3.4	15.2	0.945	0.602
Mamba-YOLO [21]	12.86	50.7	0.938	0.600
Ours	19.02	43.9	0.949	0.635

**Table 13:** External UAV benchmark results on VisDrone2019-DET. The lower block reports values quoted from the corresponding publications and was not re-trained under our protocol.

Method	Params (M)	GFLOPs	mAP_50_	mAP_50-95_
*Re-evaluated under the unified protocol*				
Faster R-CNN (ResNet-50) [26]	41.2	134.8	0.266	0.146
RT-DETR [54]	32.82	109.9	0.359	0.178
Mamba-YOLO [21]	12.86	50.7	0.433	0.255
YOLOv8n baseline	3.01	8.1	0.324	0.179
YOLOv11n baseline	2.59	6.5	0.310	0.179
YOLOv8n + proposed	19.02	43.9	**0.441**	**0.272**
YOLOv11n + proposed	9.09	37.7	0.360	0.206
*Published VisDrone results from recent UAV-oriented detectors*				
YOLOSO [36]	3.56	12.4	0.373	0.220
DPFS-YOLO [37]	2.97	14.7	0.384	0.228
EFSL-YOLO [38]	8.90	28.9	0.407	0.243

**Table 14:** Class-wise safety-oriented precision, recall, and F1 on the fixed 1625-image validation set.

Category	Precision	Recall	F1
person	0.961	0.940	0.950
helmet	0.942	0.913	0.927
insulated clothing	0.924	0.881	0.902
insulated gloves	0.861	0.803	0.831
insulated cover	0.903	0.872	0.887
insulated blanket	0.884	0.842	0.862
telegraph pole	0.952	0.931	0.941
cross arm	0.932	0.902	0.917

**Table 15:** Scale-stratified AP50 on the fixed 1625-image validation set.

Configuration	Small	Medium	Large
YOLOv8n	0.642	0.861	0.934
YOLOv8n + MSSF	0.681	0.873	0.938
YOLOv8n + FreqAFU	0.667	0.869	0.936
YOLOv8n + Mamba+BiNet	0.674	0.879	0.941
YOLOv8n + proposed	0.713	0.892	0.946

## Data Availability

The source code and the self-collected UAV dataset used in this study will be made publicly available at https://github.com/gfjgfjgfj/UAV (accessed on 13 October 2026). The repository will include the dataset annotations, benchmark partition information, model configurations, and training and evaluation scripts associated with the reported experiments.
